# Pb^2+^ Responsive Cu-In-Zn-S Quantum Dots With Low Cytotoxicity

**DOI:** 10.3389/fchem.2022.821392

**Published:** 2022-02-14

**Authors:** XiaoLe Han, Fan Yu, JiaWen Lei, Jiahua Zhu, HaiYan Fu, JunCheng Hu, Xiao-Long Yang

**Affiliations:** ^1^ Key Laboratory of Catalysis and Energy Materials Chemistry of Ministry of Education & Hubei Key Laboratory of Catalysis and Materials Science, South-Central University for Nationalities, Wuhan, China; ^2^ The Modernization Engineering Technology Research Center of Ethnic Minority Medicine of Hubei Province, School of Pharmaceutical Sciences, South-Central University for Nationalities, Wuhan, China

**Keywords:** CuInZnS quantum dots, Microcalorimetry, *IC*_50_, Fluorescent sensor, Pb^2+^, Ion detection

## Abstract

Water-soluble Cu-In-Zn-S quantum dots (CIZS QDs) with orange fluorescence have been synthesized with a glutathione (GSH) as stabilizer via facile a one-step hydrothermal method. The optimal reaction conditions of CIZS QDs including temperature, time, pH, and the molar ratios of precursors were studied. TEM results indicate that the aqueous-dispersible CIZS QDs are quasi-spherical, and the average diameters are 3.76 nm with excellent fluorescent stability. Furthermore, the cytotoxicity of CIZS QDs was investigated by the microcalorimetry combining with TEM and the *IC*_50_ was 10.2 μM . CIZS QDs showed a promising perspective in applications such as a fluorescent probe for bioimaging and biolabeling due to the low cytotoxicity and good biocompatibility. Moreover, the CIZS QDs can distinguish Pb^2**+**^ ion from other ions, offering great potentials in lead ion determination in drinking water. According to the results of UV, XRD, FL, PL, and ITC methods, the mechanism of CIZS QDs-Pb^2+^ assay is due to hydrogen bonding or van der Waals forces in the formation of Pb^2+^ and CIZS QDs.

## Introduction

Quantum dots (QDs), also known as semiconductor nanocrystals, generally have a particle size of 2–10 nm (Liu et al., 2018; Bai et al., 2019). Due to the strong quantum confinement effect, their continuous band structure becomes a discrete energy level structure (Tian et al., 2020), giving QDs unique optoelectronic properties and causing the interests of researchers (Rani et al., 2020; Adel et al., 2021). Especially, the quaternary system QDs are the most widely studied and relatively mature quaternary semiconductor. For instance, Cu-In-Zn-S (CIZS) QDs are not only having a global rich composition of non-toxic elements, but also with direct band gap covering the best energy range for photovoltaic applications (Chen et al., 2017; Chen et al., 2018; Liu et al., 2020). In addition, the introduction of Zn ions in the ternary system CIS or CISe makes the CIZS or CISe system have good stability, narrow emission line width, wide spectrum adjustable range, and high photoluminescence quantum yield (PLQYs) (Xu et al., 2017; Bai et al., 2019). Due to their unique and excellent optical properties, quaternary system QDs are often used in light-emitting solar concentrators (LSCs), bioimaging and light-emitting diodes, sensors, and other fields (Deng et al., 2012; Niu et al., 2018; Wu et al., 2018; Hu et al., 2019). At the same time, QDs are considered to be very ideal materials for preparing fluorescent probes because of their high photoluminescence efficiency and large stokes shift (Zhao et al., 2021). In this regard, for different types of QDs, their unique properties are appropriately used for application research such as the labeling and detection of ions and other substances (Gui et al., 2017; Meng et al., 2020).

In this paper, the synthesized CIZS QDs with low cytotoxicity can selectively detect lead ions. Lead is a heavy-duty, non-ferrous metal material with corrosion resistance due to its low melting point, good corrosion resistance, good X-ray impermeability, good plasticity (Bonnassieux et al., 2021; Jin et al., 2021), and widely used in plate and pipe processing (Singh et al., 2018). In addition, it is also used in industrial fields such as the chemical industry, cable manufacturing, battery production, and radiation protection (Chibowska et al., 2016; Singh et al., 2018; Bai et al., 2019). There is no doubt that lead is also one of the three major heavy metal pollutants. It is a heavy metal element that seriously harms human health. Lead in the human body mainly comes from food intake and drinking tap water. Most lead is stored in the bones, and only about 10% flows with the blood circulation distributing to various tissues and organs throughout the body, affecting the functions of red blood cells, the brain, the kidneys, and the nervous system (Chibowska et al., 2016). Especially after infants and young children absorb lead, more than 30% of the lead will stay in the body, affecting their growth and intellectual development (Senut et al., 2012). Therefore, researchers have developed many methods to detect Pb^2+^ in the past few decades to study the impact of lead on biology and the environment. Among them, the fluorescent probe method has attracted wide attention of researchers due to its high sensitivity, strong selectivity, simplicity, and low power consumption. In this paper, using glutathione as stabilizer and nitrate as precursor, hydrophilic CIZS QDs were synthesized by the one-step hydrothermal method with good biocompatibility and high fluorescence stability. According to the micro-calorimetric technique, the biological effect of CIZS QDs on *S. cerevisiae* was investigated and its low cytotoxicity is proved. What is more, in the selective detection of specific metal ions, high-sensitivity detection of Pb^2+^ is realized. In addition, we also used isothermal titration calorimetry (ITC) experiment, TEM, and fluorescence lifetime experiment to explore the mechanism of interaction between QDs and lead ions, which laid a good foundation for the design and application of fluorescent probes.

## Materials and Methods

### Chemicals and Reagents

Copper nitrate (Cu(NO_3_)_2_) was purchased from Sinopharm Chemical Reagent Co., Ltd. (Shanghai, China). Sodium sulfide (Na_2_S·9H_2_O) was purchased from Shanghai Ling Feng Chemical Reagent Co., Ltd. (Shanghai, China). Zinc nitrate (Zn(NO_3_·6H_2_O)_2_, 99.99%), glutathione (reduced) (GSH, 98%), and sodium hydroxide (NaOH, AR) were purchased from Shanghai Aladdin Bio-Chem Technology Co., Ltd. Indium nitrate (In(NO_3_)_3_, 99.99%) was bought from Shanghai Macklin Biochemical Co., Ltd.

Phosphatebuffer saline (PBS, pH = 7.4), and yeast extract peptone dextrose (YPED) medium were prepared by dissolving yeast extract (10 g), peptone (10 g), and glucose (20 g) in DI water (1.0 L) at natural pH, which was then sterilized under high-pressure steam at 120°C for 30 min. The water used in the experiment is deionized distilled. All the chemicals were of analytical grade and used without further purification.

### Instrumentation

The photoluminescence (PL) emission and ultraviolet-visible (UV-Vis) absorption spectrums were measured by the fluorescence spectrophotometer (HITACHI F-4600) and ultraviolet-visible spectrophotometer (HITACHI U-3900H), respectively. Transmission microscopy (TEM, TECNAI G220 S-TWIN) was used to obtain the shape and size distribution of CIZS QDs. XPS spectra was acquired via X-ray photoelectron spectroscopy (MULTILAB2000) for determining the elementary composition. X-ray diffraction (XRD) measurement was carried out using X-ray diffractometer (D8 ADVANCE) to analyze the crystalline structure of CIZS QDs. The fluorescence lifetime of CIZS QDs was measured by photoluminescence spectrometer (FLS 1000) to obtain the average fluorescence lifetime of QDs and the decay model of fluorescence intensity.

### Synthesis of CIZS QDs

The CIZS QDs were synthesized by a simple hydrothermal method. Typically, 1.9 mg of Cu(NO_3_)_2_ (10^−5^ M), 30.08 mg of In(NO_3_)_3_ (10^−5^ M), 14.87 mg of Zn(NO_3_)_2_·6H2O (5 × 10^−5^ M), and 0.2151 g of GSH were mixed with 48 ml of water. Then, 0.1680 g of Na_2_S·9H_2_O was dispersed in 2 ml of water and slowly poured into the mixture. Subsequently, the NaOH solution (1 and 0.1 M) was used dropwise to adjust the pH of the mixture to 9.0 under vigorous stirring. Then the mixture was place into a three-necked flask followed by heating 120°C under an open-air atmosphere using a condenser. At various time intervals, aliquots of reaction solution were withdrawn, and the reaction was stopped after 3 h. After the reaction solution was cooled to room temperature, CIZS QDs were precipitated by adding twofold ethanol. Then the precipitates were dispersed in water and freeze-dried thoroughly for further use. After that, by changing the experimental variables such as reaction temperature, reaction pH, reaction time, and precursor molar ratio, the fluorescence intensity of aqueous CIZS QDs were investigated on the different conditions.

### TAM Air Experiment

Microcalorimetry is the science of measuring heat changes from chemical reactions or physical events. In this experiment, the metabolism thermogenic curves of *S. cerevisiae* in YPED medium with different concentrations of CIZS QDs at 30°C were investigated. The specific experimental process was as follows: First, *S. cerevisiae* was cultured in YPED medium for 10 h at 30°C. Second, 50 ml of sterilized YPED medium was mixed with 500 μl of *S. cerevisiae* solution, and then separated into 8 ampoules via pipette and added 5 ml yeast liquid into each ampoule. Third, different amounts of CIZS QDs (0, 0.8, 1.2, 1.6, 2.4, 3.2, 4.8, 9.6 μM) were added into these ampoules, respectively. Then all these ampules were sealed and oscillated thoroughly. Finally, transfer the packaged ampoule to isothermal microcalorimeter (TAM air) for measuring heat changes of *S. cerevisiae* incubated with different concentrations of CIZS QDs. After the experiments, the data were analyzed. All the experiments were repeated three times.

### Microscopic Experiment

*S. cerevisiae* was incubated with CIZS QDs (1.6, 4.8 μM) in YEPD at 30°C for 6 h as the absorbance rose to 0.8 (about 360 min) at 600 nm (*A*_600_) for laser scanning confocal and TEM.

### Isothermal Titration Calorimetry (ITC) Experiment

Isothermal titration calorimetry (ITC) is a technique for measuring the heat of titration reaction between the titrant and the titrant under the condition that the sample and the reference are at the same temperature. Like ordinary acid-base titration, there will be a heat change after each titration, and ITC can just capture this heat change. Under the condition of 298.15 K, the titrated substance is CIZS QDs (2.3 × 10^−4^ M), the titrant is Pb^2+^ solution (10^−7^ M), and the total amount of titrant is 50 µl, which is instilled 25 times, 2 µl each time. Titration interval is 200 s.

## Results

### Synthesis and Characterization of CIZS QDs

#### Optical Properties

CIZS QDs were prepared by a hydrothermal reaction between the nitrate salts of the corresponding metals and sodium sulfide as sulfide precursor at 120°C. In this study, we investigated the effects of reaction temperature, reaction time, pH value of reaction, and the molar ratio of different precursors on the fluorescence intensity of the synthesized QDs to confirm the optimum reaction condition. The related results are shown in Supplementary Figure S1. According to the experiments, the PL intensity of CIZS QDs was the strongest when the molar ratios of precursors were Cu:In:Zn:S = 1:10:70:70 and Cu:Zn = 1:5. Meanwhile, we found the optimal reaction time was 3.0 h, reaction temperature was 120°C, and pH value of the reaction was 9.00.

**FIGURE 1:**
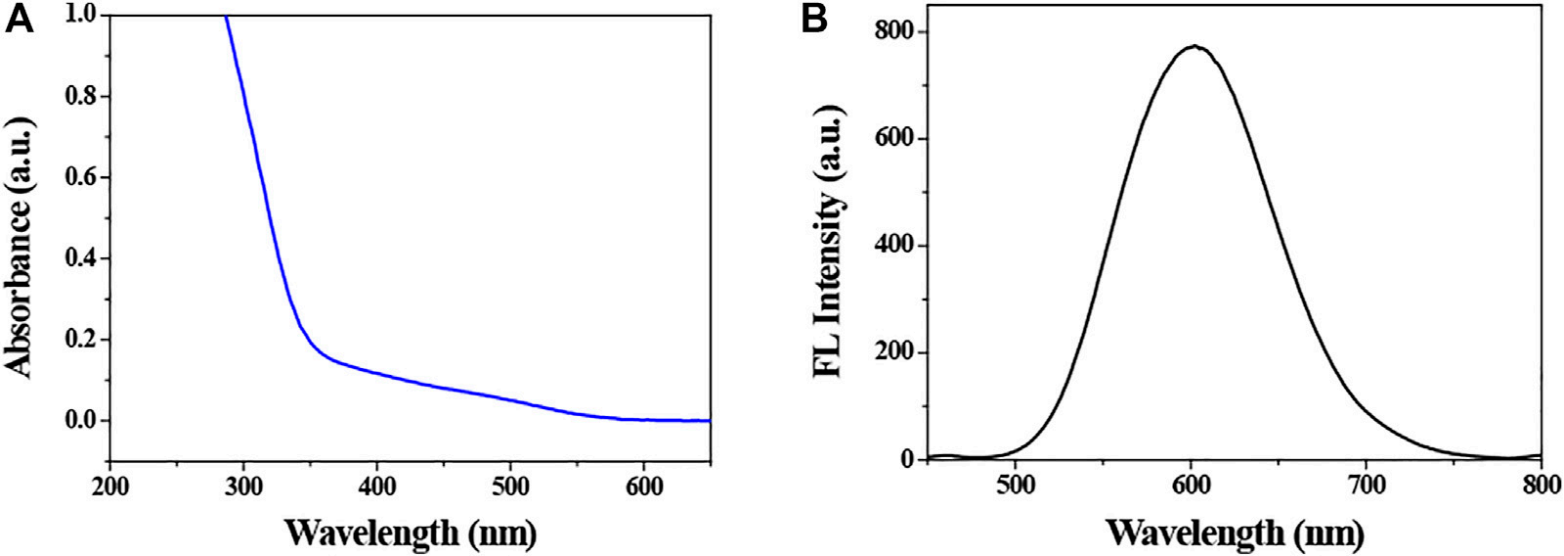
**(A)** Absorption and **(B)** photoluminescence spectrums of CIZS QDs (the concentration is 2.3 × 10^−4^ M). The related absorption and fluorescence peak positions were 365 and 603 nm, respectively.

The synthesized QDs were characterized by fluorescence spectroscopy and UV-vis spectrophotometer. The fluorescence emission spectrum and UV-visible spectrum of QDs are shown in Figure 1. It indicates that the position of emission and absorption peaks is at 603 and 365 nm, respectively. In addition, the fluorescence of QDs was orange under the irradiation of 365 nm ultraviolet lamp, which was enhanced gradually with increasing the reaction time, and reached a maximum at 3.0 h, followed by a slight decrease. It suggested that the PL intensity of CIZS QDs reached the maximum when the reaction time was 3.0 h. Therefore, the CIZS QDs used in the following tests were all samples that reacted at 3.0 h.

Fluorescence quantum yield is an indispensable analytical quantity for the characterization of synthetic quantum dots, which was obtained by photoluminescence spectrometer. The results showed that the PL quantum yield is about 9.18%, which was obtained after three repeated tests. Meanwhile, the quantum yield of CIZS QDs obtained from a photoluminescence spectrometer was reconfirmed with the data calculated with rhodamine B as control. The quantum yield of CIZS QDs was calculated according to Eq. 1.

$$Y_{u}=Y_{s}\times \frac{F_{u}}{F_{s}}\times \frac{A_{s}}{A_{u}}$$ (1)

where *Y*_*u*_ is the quantum yield of CIZS QDs and *Y*_*s*_ is the quantum yield of rhodamine B. *F*_*u*_ and *F*_*s*_ are the integrated fluorescence intensity of CIZS QDs and rhodamine, respectively. A_u_ and A_s_ represent the maximum absorbance value of CIZS QDs and rhodamine B, respectively. Besides, according to the literature report (Guo et al., 2013), the PL quantum yield of rhodamine B is 89%. We can speculate that the PL quantum yield of CIZS QDs was 9.16%, which was basically the same as the data obtained from the above test.

#### Morphology and Structure Characterization of CIZS QDs

**FIGURE 2:**
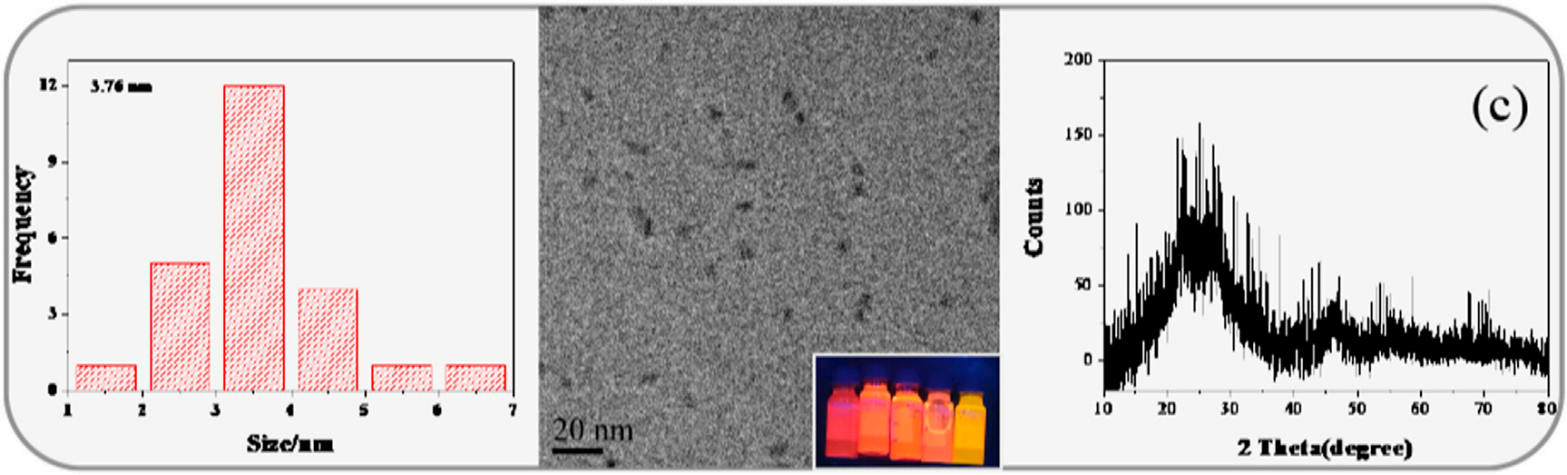
**(A)** The corresponding histograms of size distribution of CIZS QDs; **(B)** TEM image of CIZS QDs, inset: digital imaging under different Cu-Zn molar ratio UV lamps; **(C)** XRD pattern of CIZS QDs.

**FIGURE 3:**
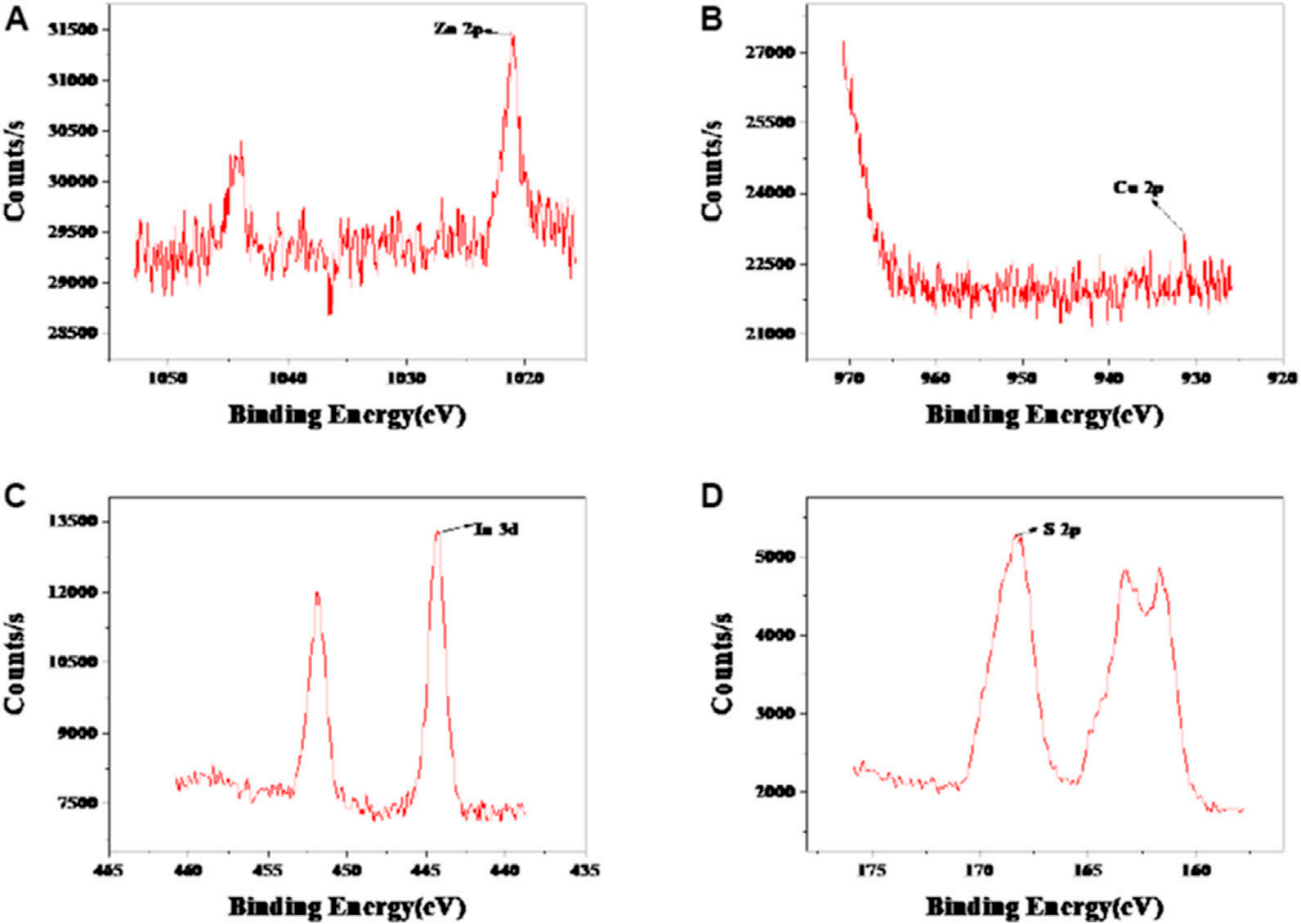
XPS of spectrum of CIZS QDs. **(A)** Zn 2p spectrum; **(B)** Cu 2p spectrum; **(C)** In 3 d spectrum; and **(D)** S 2p spectrum of CIZS QDs.

The morphology, particle size, and dispersion of the synthesized QDs can be observed by means of microscope. The TEM observation of CIZS QDs is in Figure 2B. The wide-field TEM images of CIZS QDs revealed that QDs have a good monodispersity and quasi-spherical morphology. As shown in Figure 2A, the average particle size of the CIZS QDs was calculated to be 3.76 nm. Figure 2C was the XRD pattern for a sample of CIZS QDs with the optimal reaction condition. It showed that the XRD pattern of CIZS QDs consist of the characteristic peaks of the zinc blended (cubic) structure (Park and Kim, 2011). In addition, it matched well the pattern of the CuInS_2_ bulk (JCPDS:47-1372) (Park and Kim, 2011). The diffraction peaks were broadened due to the finite particle size of CIZS QDs. Moreover, as shown in Figure 3, the XPS pattern of CIZS QDs demonstrated the existence of Cu, In, Zn, and S of quaternary CIZS QDs, which were consistent with the fact that they were obtained from the XRD pattern of CIZS QDs. The elementary composition of Cu, In, Zn, and S of CIZS QDs was 0.10, 0.32, 0.39, and 5.76%, respectively. Thus, the approximate ratio of these elements was Cu:In:Zn:S = 1:3:4:57.

### The Investigation of Cytotoxicity of CIZS QDs

#### The Metabolic Growth Rate Constant and Half Inhibitory Concentration (*IC*_50_)

**FIGURE 4:**
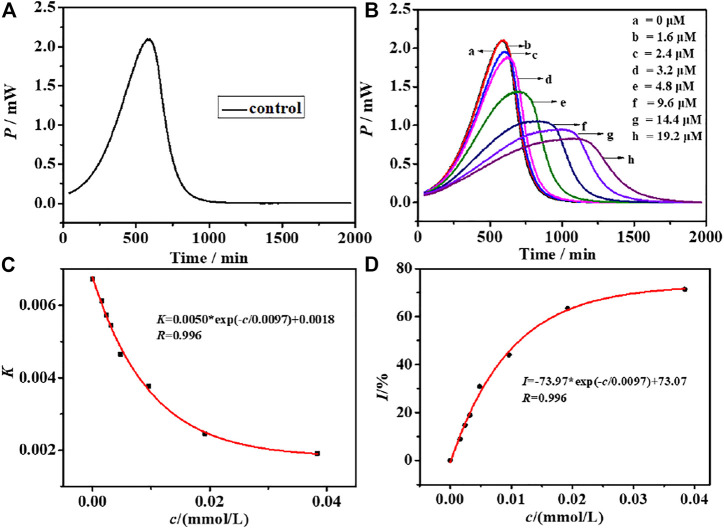
**(A)** Growth thermogenic curve of *S. cerevisiae* without CIZS QDs and **(B)** affected by CIZS QDs with different concentrations at 30°C; relationship between the CIZS QDs with different concentrations and **(C)** growth rate constant *k*; **(D)** fitting graph of the relationship between CIZS concentration and inhibition ratio.

To evaluate the cytotoxicity of CIZS QDs, the effects of CIZS QDs on *S. cerevisiae* were investigated by using a TAM air micro-calorimeter and TEM. *S. cerevisiae* was selected as a unicellular eukaryotic model organism because it has been widely used as in the toxicological evaluation of chemicals (Chouhan et al., 2014). The typical thermogenic power-time (*p*-t) curve of *S. cerevisiae* as a control group without CIZS QDs was shown in Figure 4A. Comparing to the thermogenic curves of *S. cerevisiae* with different concentrations of CIZS QDs (Figure 4B), we could find the metabolism of *S. cerevisiae* was changed regularly and the heat output in the metabolism process decreased gradually with the increase of concentration of CIZS QDs. It suggested that CIZS QDs have a certain effect on the growth and metabolism of yeast. According to the thermokinetic Equation 3, the data are analyzed and the related thermodynamic and kinetic information about the multiplying metabolism are listed in Table 1.

$$P_{t}=P_{0}\text{exp}\left(kt\right)$$ (2)

Or

$$\ln P_{t}=\ln P_{0}+k_{t}$$ (3)

where *t* is the incubation time, *P* is the power output at time *t*, *P*_0_ is the power at time *t* = 0, and *k* is the growth rate constant calculated from the slope of the semi-logarithm of the exponential phase (Han et al., 2019). As shown in Figure 4C, the relationship between growth rate constant *k* and concentration of CIZS QDs was fitted and there was a good exponential relationship between them. Then we calculated the inhibitory ratio (*I*) and the half inhibitory concentration (*IC*_*50*_) by formula. The inhibitory ratio is a particularly important parameter to detect the biological toxicity of QDs, which can be calculated through Eq. 4:

$$I=\left[\frac{k_{0}-k_{c}}{k_{0}}\right]\times 100\%$$ (4)

where *k*_0_ is the rate constant of the control and *k*_*c*_ is the rate constant for microbes inhibited by an inhibitor at a concentration *c* (Han T. et al., 2020). Similarly, with the addition of concentration of CIZS QDs, the inhibitory ratio (*I*) increased gradually, as seen in Figure 4C. When the inhibitory ratio (*I*) is equal to 50%, the half inhibitory concentration (*IC*_*50*_) was obtained, which could represent the inhibition capability of the compound quantitatively. The smaller the value of *IC*_*50*_, the stronger its inhibitory activity. According to the fitted relationship between the inhibitory ratio (*I*) and the concentration of CIZS QDs, the value of *IC*_*50*_ was equal to 10.2 μM (Table 1). According to the data studied by our group previously (Han et al., 2019; Han XL et al., 2020) (Supplementary Tables S1, S2), it suggested that CIZS QDs had less toxicity. The results of electron microscopy also confirmed this conclusion.

**TABLE 1:** Parameters of *S. cerevisiae* growth at different concentrations of CIZS QDs.

QDs	concentration of QDs	rate constant	Deviation from the linear relationship	The maximum of power output	The total of heat	the inhibitory ratio	half inhibitory concentration
	[μM ]	[10^−3^min^−1^]		[mW]	[J]	[%]	[μM ]
CIZS QDs	0	6.72	0.999	2.10	0.791	0	**10.2**
	1.60	6.62	0.996	2.11	0.801	8.93	
	2.40	5.73	0.996	1.96	0.765	14.73	
	3.20	5.45	0.991	1.88	0.783	18.90	
	4.80	4.65	0.992	1.44	0.786	30.80	
	9.60	3.76	0.994	1.06	0.746	44.05	
	14.40	2.46	0.995	0.95	0.781	64.40	
	19.20	1.92	0.991	0.83	0.786	71.43	

#### Morphological Changes of *S. cerevisiae* Treated With CIZS QDs

**FIGURE 5:**
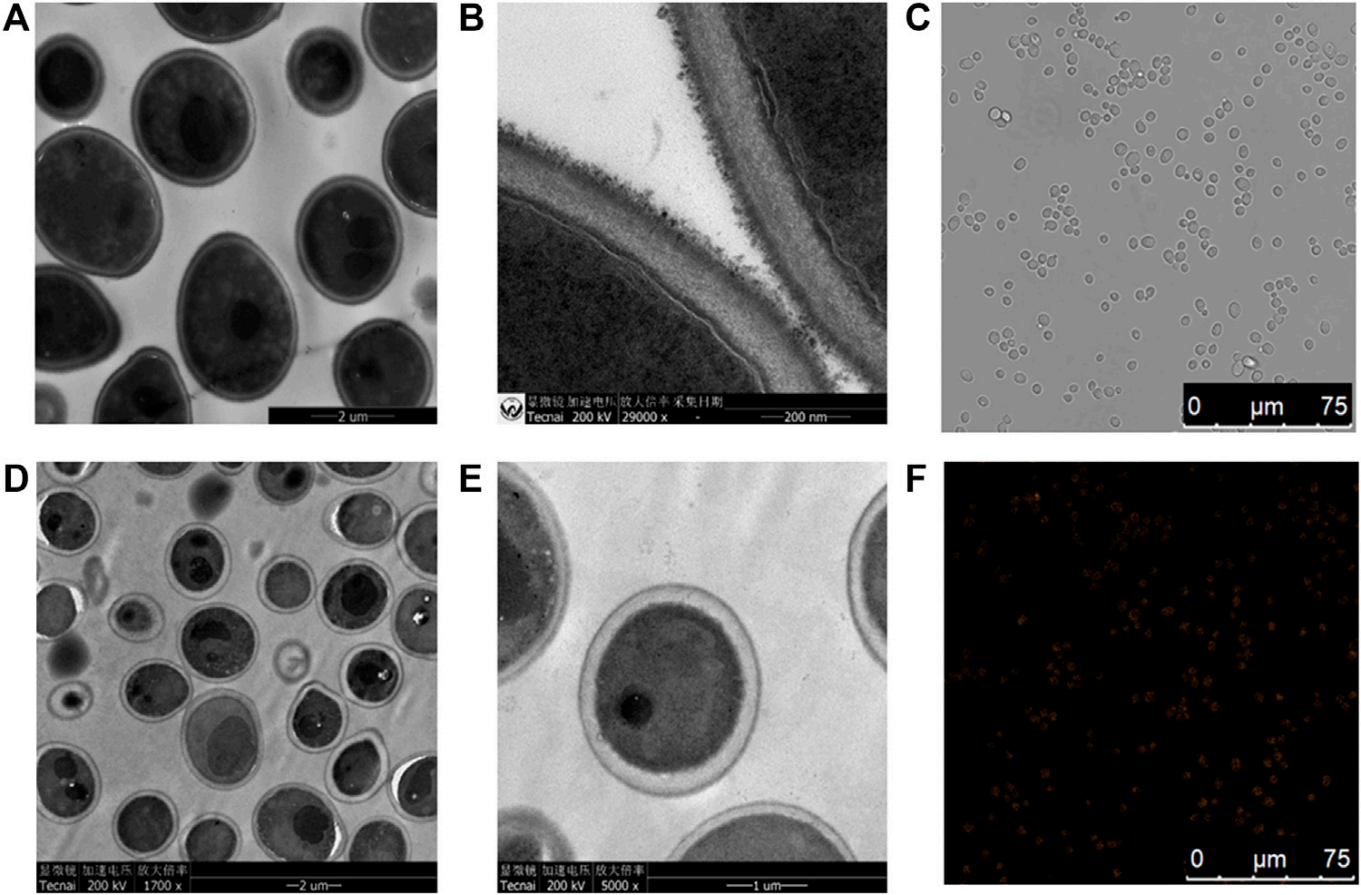
TEM images of a thin section of yeast cells after incubation. **(A,B)** Control group of yeast cells without CIZS QDs; **(D,E)** test group of yeast cells of 5 h incubation with CIZS QDs; **(C,F)** confocal images of yeast cells incubated with CIZS QDs under the excitation at 405 nm. The concentrations of CIZS QDs were 1.6 μM.

Just as shown in Figure 5, TEM images of *S. cerevisiae* treated with a certain concentration of CIZS QDs (Figures 5D,E) were found to have some differences of morphology in comparison with the control groups without CIZS QDs (Figures 5A,B). The control cell morphology is clear, and the cell wall is intact. Comparing with another two images representing the morphology of *S. cerevisiae* treated with CIZS QDs, some white vacuoles appeared in the cytoplasm. However, most of them remained intact. This phenomenon revealed that CIZS QDs had a negligible effect on the morphology of *S. cerevisiae.* Meanwhile, we found CIZS QDs could be internalized into the yeast cells by using laser scanning confocal microscopy. The yeast cells have been incubated with CIZS QDs for 6 h to analyze the intracellular uptake of QDs. Figures 5C,F represented the bright-field and dark-field images of yeast cells incubated with CIZS QDs, respectively. In Figure 5F, the orange fluorescence of CIZS QDs could be observed in yeast cells after being incubated with *S. cerevisiae*, which indicated that the CIZS QDs can be applied in bioimaging. We speculated CIZS QDs have a good potential application in bioimaging due to the low cytotoxicity and fluorescent stability.

### CIZS QDs were Developed for Pb^2+^ Assay and Promising Quenching Mechanism

#### The Influence of Pb^2+^ on the Structure of CIZS QDs

**FIGURE 6:**
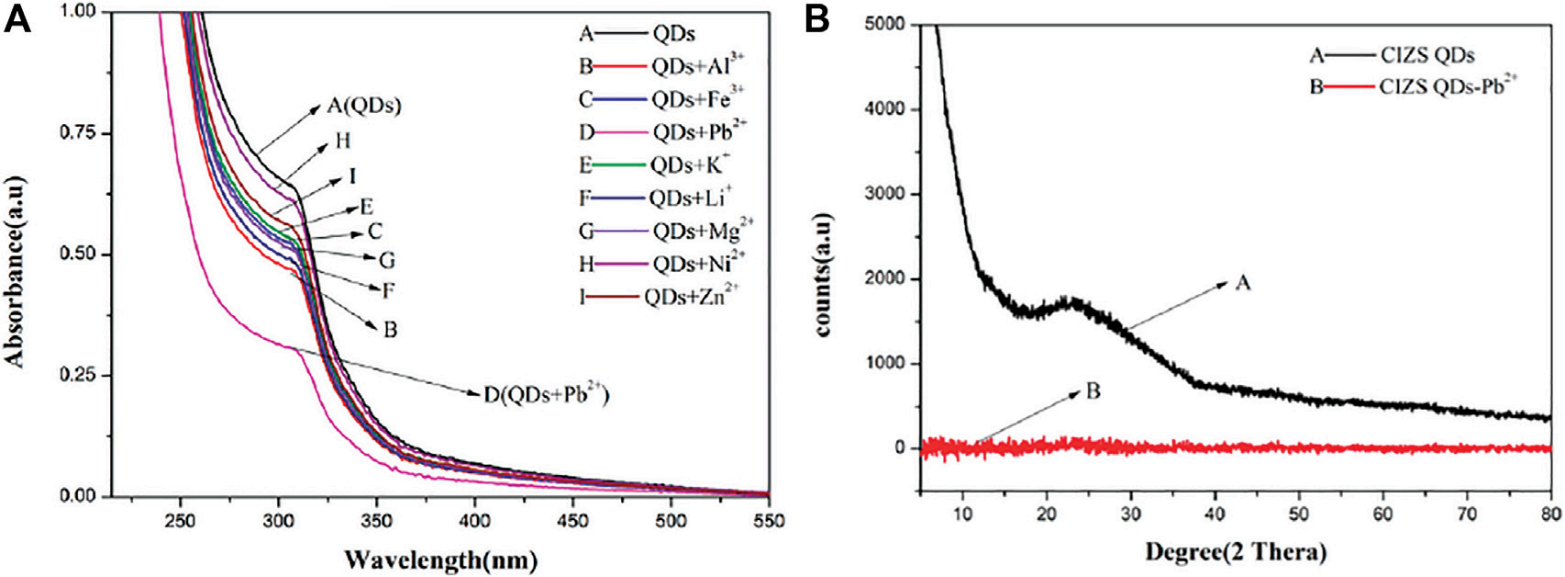
**(A)** The ultraviolet absorption curves of CIZS QDs in the presence of different metal ions. **(B)** Comparison of X-ray diffraction patterns of CIZS QDs before and after Pb^2+^ addition.

UV-vis absorption measurement is a sensitive method to explore the structural changes and to know the complex formations (Bashir et al., 2021). Figure 6A is the UV absorption spectrum of CIZS QDs only (A) and the absorption of CIZS QDs with the eight diverse kinds of metal cations (B-I). It is shown that the absorbance of CIZS QDs decreased with joining the same concentration of different metal cations to varying degrees, especially lead ions, leading to a most significant reduction. Meanwhile, after joining Pb^2+^, CIZS QDs have a slight red shift phenomenon. We concluded that CIZS QDs can detect Pd^2+^ sensitively due to forming complexes of Pb^2+^- CIZS QDs and speculated the structure of CIZS QDs has a certain influence. The XED data confirmed our prediction. Figure 6B was an obvious wide peak in the XRD pattern of CIZS QDS in the range of 10–80°, and the peak position was about 23°. According to the Bragg equation (2d⁡sin⁡θ=nλ) , the crystal plane distance at 23° was 0.29 nm. When Pb^2+^ was added to CIZS QDs, there was no obvious peak in the XRD pattern. It can be proved that after adding Pb^2+^ to CIZS QDs, the original crystal structure of CIZS QDs has been changed (De Acha et al., 2019; Shu et al., 2020; Vaishanav et al., 2021).

#### Fluorescence Detection of Pb^2+^

**FIGURE 7:**
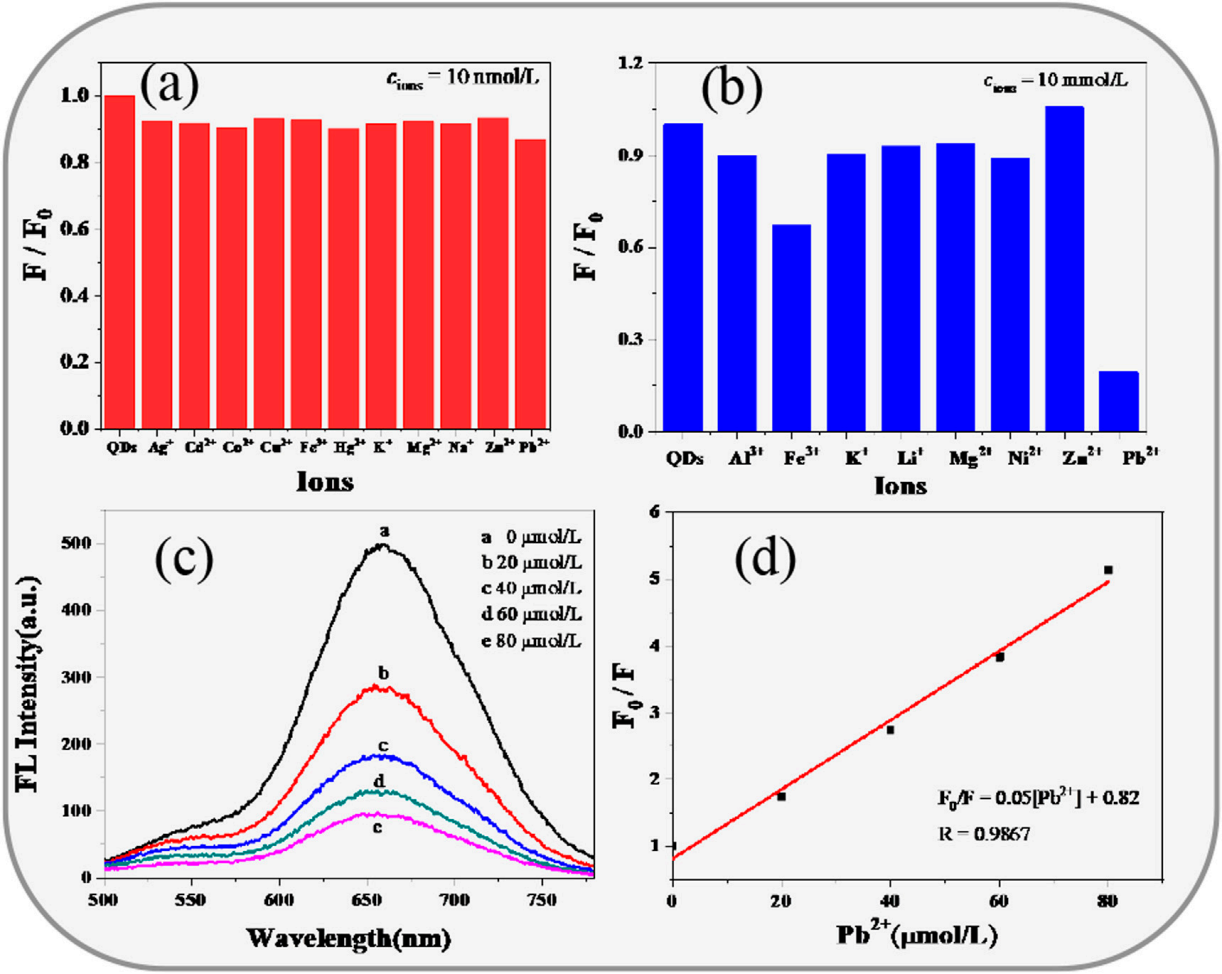
Fluorescence detection of Cu^2+^ by CIZS QDs. the relative fluorescence emission intensity at 603 nm of CIZS QDs in the presence of different metal ions, **(A)** the concentration of the metal cation is 10 nM and **(B)** 10 mM; **(C)** fluorescence emission quenching spectra of CIZS QDs with different concentrations of Pb^2+^, the concentrations of Cu^2+^ are 0, 20, 40, 60, and 80 μM; **(D)** the linear relationship between the fluorescent intensity ratios of F_0_/F and the concentration of Pb^2+^; all the experiments were conducted three times in parallel.

We further attempted to use CIZS QDs with low toxicity and good fluorescence stability as an ion detection probe. By measuring the PL intensities of CIZS QDs in the presence of 11 other kinds of metal ions, we found no significant difference at the concentration of all metal ions which is 10 nM, respectively, from Figure 7A. However, as the concentration of metal ions increased to 10 mM, it could be seen that Pb^2+^ had the largest quenching effect on the fluorescence intensity of CIZS QDs (in Figure 7B). It revealed the PL emission of orange emitting CIZS QDs could be selectively quenched by Pb^2+^ without extra surface modification. Furthermore, we carried about the experiment related to the fluorescence spectra of CIZS QDs quenched by the addition of different concentrations of Pb^2+^ (in Figure 7C). It was illustrated that the fluorescence of CIZS QDs can be selectively quenched by Pb^2+^ to a great extent. All the data were analyzed according to the Stern-Volmer Eq. 5.

$$\frac{F_{0}}{F}=1+K_{D}\left[P_{b}^{2+}\right]$$ (5)

where *F*_*0*_ and *F* are PL intensity of CIZS QDs with and without the quenchers [Pb^2+^], *K*_*D*_ is the Stern-Volmer constant. The relationship between relative PL intensity (F_0_/F-1) of CIZS QDs and the concentration of quencher [Pb^2+^] is illustrated in Figure 7D. From the linear Stern-Volmer plot, the calculated K_D_ value is 0.052 × 10^6^ M^−1^, and the LoD is estimated using the formula $3S_{b}/k$ , which comes out to be 5.18 × 10^−7^ M for Pb^2+^. Where *k* is the slope of the S-V plot and *S*_b_ denotes the standard deviation (Vaishanav et al., 2021; Wang et al., 2021). Furthermore, the quenching rate constant $k_{q}$ , calculated by Eq. 6, was obtained to be 1.2 × 10^10^mol^−1^s^−1^.

$$K_{D}=k_{q}\cdot \tau_{0}$$ (6)

where τ_0_ denotes the lifetime of CIZS QDs in the absence of a quencher. The maximum value of $k_{q}\;$ for static collisional quenching is about 1∼2 × 10^10^mol^−1^s^−1^ (Kumar et al., 2021). The calculated value of $k_{q}$ is within the range of the value indicating that the quenching is caused by the static due to complex formation in the ground state (Passos et al., 2020; Han et al., 2020; You et al., 2020). To further understand the mechanism of PL quenching by Pb^2+^ ions, the change in average lifetime provides us with a clear thought.

#### Effect of Pb^2+^ on the Fluorescence Lifetime of CIZS QDS

The instantaneous fluorescence lifetime of CIZS QDS and the instantaneous fluorescence lifetime after adding Pb^2+^ were measured by instantaneous fluorometer. The PL decay curves of CIZS QDs were fitted by the triexponential function equation:

$$I_{t}=I_{0}+A_{1}\exp\left(-\frac{t-t_{0}}{\tau_{1}}\right)+A_{2}\exp\left(-\frac{t-t_{0}}{\tau_{2}}\right)+A_{3}\exp\left(-\frac{t-t_{0}}{\tau_{3}}\right)$$ (7)

where τ_1_, τ_2_, and τ_3_ denote the photoluminescent fluorescence lifetime of CIZS QDS, and A_1_, A_2_, and A_3_ denote the relative weight of the decay component at t = 0. According to the fitting results in (Supplementary Figure S2), the values of A_1_, A_2_, and A_3_ accounted for 5.9%, 6.5%, and 88.0%, respectively. Besides, τ_1_, τ_2_, and τ_3_ accounted for 0.89, 8.88, and 0.89 µs, respectively. Among them, τ_1_ and τ_3_ are associated with short-lived decay, while τ_2_ stands for long-lived decay. The average lifetime was calculated from the following equation (Sun et al., 2021):

$$\tau_{av}=\sum A_{i}\tau_{i}^{2}/\sum A_{i}\tau_{i}$$ (8)

The average life ( $\tau_{av}$ ) of CIZS QDS can be calculated as 4.2 µs. The PL decay curves of CIZS QDs-Pb^2+^ fitted by the triexponential function were shown in Supplementary Figure S2B, A_1_, A_2_, and A_3_ accounted for 2.7%, 18.2, and 79.1%, respectively; τ_1_, τ_2_, and τ_3_ accounted for 9.21, 0.21, and 0.21 µs, respectively. Besides, the average life ( $\tau_{av}$ ) of CIZS QDS-Pb^2+^ can be calculated as 2.7 µs. Through the comparison of the two sets of data before and after, the average fluorescence lifetime of the QDs after adding Pb^2+^ is slightly reduced. This also shows that CIZS QDs form a complex with Pb^2+^. In contrast, the fluorescence lifetime of CIZS QDs has changed from 4.2 to 2.7 µs with the addition of Pb^2+^. CIZS QDS-Pb^2+^ exhibit a more rapid decay of photoluminescence which is attributed to nonradiative recombination induced by the intrinsic and surface defects (Leng et al., 2014). Except the contribution of fast decay components τ_1_ increased while fast decay components τ_2_ and long decay τ_3_ component decreased for the CIZS QDS. According to the research, the fast decay component is related to nonradiative recombination associated with surface defects (Zhao et al., 2017; Liu et al., 2021; Li et al., 2021). CZIS QDs with no extra modification contain plenty of surface dangling bonds which can act as trap states for charge carriers (Wang et al., 2015). The empty orbit of the Pb^2+^ interacts with the outer electron of the S^2-^, thereby reducing the surface defects of the CIZS QDs, improving the non-radiative recombination life of CIZS. However, a number of structural defects was enhanced due to the forming complex of CIZS QDs-Pb^2+^, and thus suppressing nonradiative recombination process. In summary, the fluorescence lifetime of CIZS QDs is shortened provided good evidence for the formation complex of CIZS QDs-Pb^2+^.

#### Thermodynamic Characterization of Interactions Between CIZS QDs and Pb^2+^

Isothermal titration calorimetry (ITC) has been widely used to understand molecular interactions, which provides a direct route to the complete thermodynamic and kinetic properties of molecular interactions. Therefore, ITC is used to explore the formation mechanism of CIZS QDs-Pb^2+^ complex and obtain the thermodynamic parameters and binding affinity of the interacting process (Huang et al., 2015; Zhao et al., 2017; Li et al., 2021). The solutions of CIZS QDs and Pb^2+^ were added in the calorimeter-cell and the syringe, respectively. The results were fitted by Micro Cal PEAQ-ITC Analysis software (Nano Analyze) after deducting the dilution heats and are shown in Supplementary Figure S3. Affinity constant ( $k_{d}$ ), binding site ( n ), enthalpy change ( ΔH ), and entropy change ( ΔS ) were directly obtained from the fitting curve. The Gibbs free energy (Δ*G*) were calculated according to the formula  ΔG = ΔH−TΔS = −RTlnK (where R is the gas constant and T is the thermodynamic temperature). As shown in Table 2, the negative value of ΔG demonstrated that the combination of CIZS QDs and Pb^2+^ is a spontaneous process. The values of n and *K* for Pb^2+^ binding CIZS QDs are 0.976 and 1.654 × 10^−2^ M^−1^, respectively, manifesting the certain affinity that occurred between Pb^2+^ and CIZS QDs. Elucidating the thermodynamic parameters might help provide clues on the involvement of these forces in the conjugation process. According to the view of Ross (Jiang et al., 2019; Li et al., 2020; Shi et al., 2021), when Δ*H* ^θ^ < 0 and Δ*S* ^θ^ < 0 were associated with hydrogen bonding or van der Waals forces, the negative values obtained for both Δ*H* ^θ^ (−4.428 × 10^4^ J·mol^−1^) and Δ*S* ^θ^ (−1.144 × 10^2^ J·mol^−1^ K^−1^) in this study suggested the involvement of hydrogen bonding or van der Waals forces in the formation of Pb^2+^and CIZS QDs. The negative enthalpy change is due to the broken Pb^2+^-H_2_O bond, which is favorable for the formation of the Pb^2+^-CIZS QD complex, while the negative entropy contributes to the formation of the complex that decreases the chaos of the interaction system.

**TABLE 2:** Thermodynamic parameters obtained by ITC for the interaction between CIZS QDs and Pb^2+^.

Model	Variable	Value
Independent	K_d_ (M)	1.654 × 10^−2^
	Δ*H* (kJ·mol^−1^)	−44.28
	Δ*S* (J·mol^−1^ K^−1^)	−114.4
	*n*	0.976

## Conclusion

In summary, we synthesized a facile quaternary CIZS QDs via a one-step hydrothermal method in the usual atmosphere pressure, under the optimal reaction condition (t = 3.0 h, T = 120°C, pH = 9.00, the molar ration is Cu:In:GSH:S: n = 1:10:70:70:5). The QDs were characterized quasi-spherically, and their average diameter were 3.76 nm with excellent orange fluorescence. According to the value of *IC*_*50*_ (10.3 uM), CIZS QDs were proved to have low cytotoxicity. Meanwhile, CIZS QDs can be internalized in the yeast cell and fluorescence and stability can be seen. Without any modification, the synthesized CIZS QDs have a rapid and gentle response to Pb^2+^ ion. The fluorescence of CIZS QDs can be used to detect the Pb^2+^ ion as a fluorescent sensor, the limit of detection (LoD) is 5.18 × 10^−7^ M for Pb^2+^. According to the PL and ITC experiments, the quenching mechanism is judged to be static quenching, involving hydrogen bonding or van der Waals forces in the formation of Pb^2+^ and CIZS QDs.

## Data Availability

The raw data supporting the conclusion of this article will be made available by the authors, without undue reservation.
