# Supplementary material for: Pb2+ Responsive Cu-In-Zn-S Quantum Dots With Low Cytotoxicity
Source: Front Chem. 2022 Feb 14;10:821392. doi: 10.3389/fchem.2022.821392 (PMC8883431; doi:10.3389/fchem.2022.821392)
Supplement: Supplementary file 1 [file DataSheet1.pdf]

Supporting Information

Pb^{2+} responsive Cu-In-Zn-S Quantum Dots with Low Cytotoxicity

Xiaole Han[1,*,#], Fan Yu[1,#], Jiawen Lei[1], Jiahua Zhu[1], Haiyan Fu[2], Juncheng Hu[1] and Xiaolong Yang[2,*]

[1]Key Laboratory of Catalysis and Energy Materials Chemistry of Ministry of Education & Hubei Key Laboratory of Catalysis and Materials Science, South-Central University for Nationalities, Wuhan 430074, China

[2]The Modernization Engineering Technology Research Center of Ethnic Minority Medicine of Hubei Province, School of Pharmaceutical Sciences, South-Central University for Nationalities, Wuhan 430074, P.R. China

---

***Correspondence:** Hubei Key Laboratory of Catalysis and Materials Science, School of Chemistry and Materials Science, South-Central University for Nationalities, Wuhan 430074, China

***Corresponding authors:** Xiaole Han and Xiaolong-Yang

[#]Co-first authors: Xiaole Han and Fan Yu

E-mail address: HXL1220@hotmail.com (Xiaole Han);yxl19830915@163.com (Xiaolong Yang)

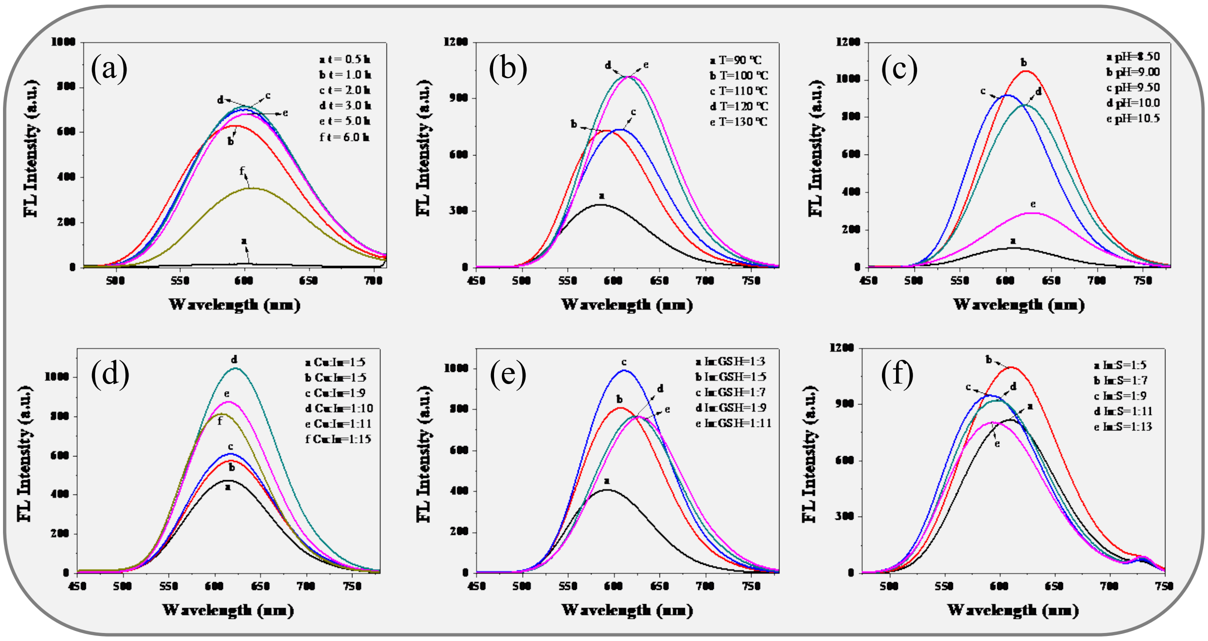

Figure S1. Effects of (A) Reaction time;(B) reaction temperature; (C) pH value; (D-F) molar ratios of Cu-In, In-GSH and In-S on fluorescence intensity of CIZS QDs under the excitation wavelength of 400 nm.

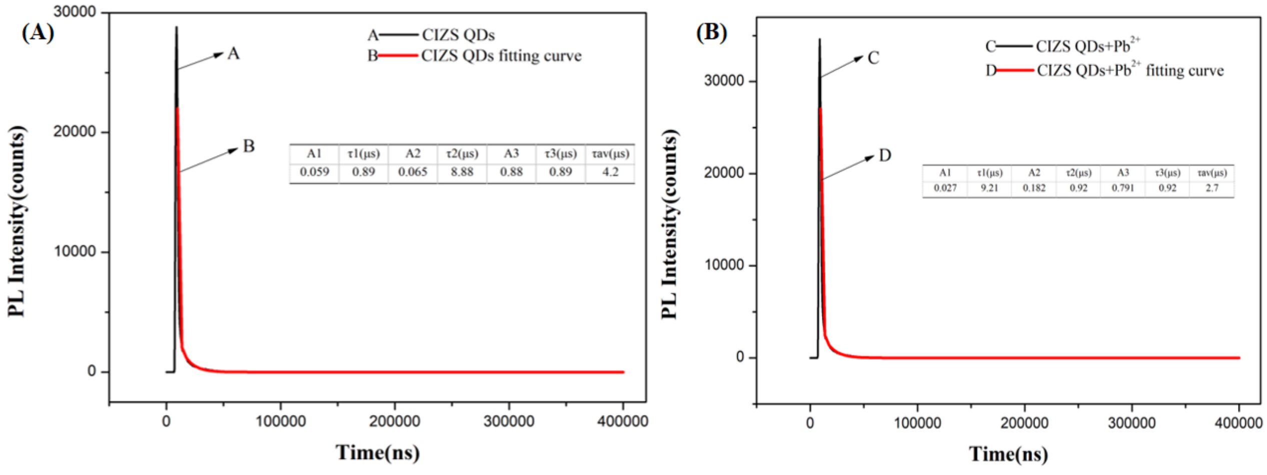

A1	τ1(μs)	A2	τ2(μs)	A3	τ3(μs)	τav(μs)
0.059	0.89	0.065	8.88	0.88	0.89	4.2

A1	τ1(μs)	A2	τ2(μs)	A3	τ3(μs)	τav(μs)
0.027	9.21	0.182	0.92	0.791	0.92	2.7

Figure S2. (A) Fitting diagram of CIZS QDS fluorescence lifetime decay. (B) Fitting diagram of fluorescence lifetime decay after adding Pb^{2+} to CIZS QDs.

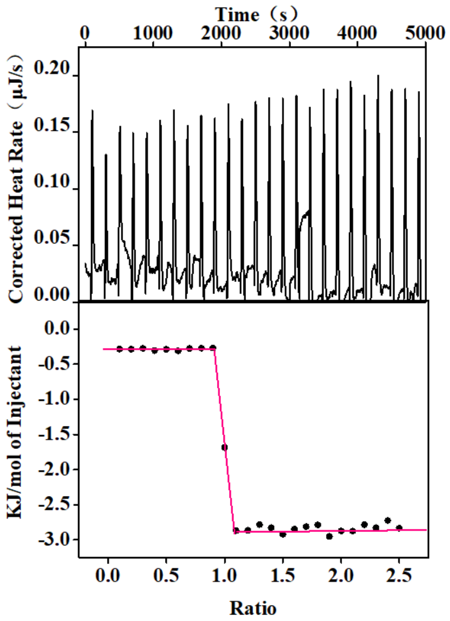

Figure S3. Isothermal titration calorimetry profiles showing complexation of QDs with Pb^{2+}. The solid line represents the best-fit plots obtained using the "one set of binding sites" model.

Table S1. The comparison of cytotoxicity of different types of QDs to *S. cerevisiae* cells (Han XL et al., 2012; Han XL et al., 2019)

QDs	O-CdTe	G-CdTe	NAC-CdTe	MPA-CdTe	GSH-CdTe	CIZS
IC_{50}[nM]	17.07	80.81	89.8	56.2	15.3	1.02×10^4

Table S2. Parameters of S. cerevisiae growth at different concentrations of QDs.

QDs	*c* [nmol·L^{-1}]	*k* [min^{-1}]	R^2	P_m [mW]	Q_{total} [J]	*I* [%]	IC_{50} [nmol·L^{-1}]
**O-CdTe**	0	0.00680	0.998	2.22	0.79	0	**17.07**
	4.9	0.00610	0.999	2.19	0.78	10.29	
	7.9	0.00540	0.997	1.69	0.84	20.58	
	11.9	0.00465	0.997	1.27	0.84	31.62	
	14.9	0.00376	0.995	1.14	0.83	44.70	
	29.7	0.00271	0.999	0.90	0.84	60.15	
	89.1	0.00005	0.997	0.62	0.85	92.05	
	198.1	0.00003	0.980	0.33	0.78	95.89	
**G-CdTe**	0	0.00728	0.998	2.35	0.81		**80.81**
	28.6	0.00766	0.999	2.31	0.78	-5.22	
	47.6	0.00725	0.997	2.34	0.81	0.41	
	61.9	0.00545	0.997	2.12	0.82	24.83	
	76.2	0.00401	0.995	1.67	0.86	44.69	
	95.2	0.00265	0.999	1.32	0.86	63.45	
	133.3	0.00107	0.997	0.93	0.85	85.24	
	209.4	0.00008	0.997	0.69	0.79	98.89	
**NAC-CdTe**	0	6.40	0.908	0.46	0.91	0	**89.8**
	38.08	7.39	0.999	0.49	1.01	-15.5	
	57.12	5.39	0.997	0.43	1.04	15.8	
	76.16	4.09	0.993	0.37	1.05	36.1	
	95.20	2.64	0.995	0.29	1.04	58.8	
	190.40	0.53	0.992	0.13	0.88	91.7	
**MPA-CdTe**	0	6.10	0.998	0.48	1.22	0	**56.2**
	6.24	5.21	0.999	0.46	1.18	14.6	
	31.20	4.20	0.997	0.39	1.28	31.2	
	93.60	1.95	0.997	0.26	1.32	68.0	
	187.20	0.87	0.995	0.20	1.22	85.7	
	312.00	0.25	0.999	0.16	1.04	95.9	
**GSH-CdTe**	0	5.82	0.998	0.48	0.86	0	**15.3**
	6.24	4.72	0.99	0.45	0.80	19.9	
	12.48	3.38	0.997	0.28	0.89	42.6	
	24.96	1.48	0.997	0.22	0.88	71.5	
	49.92	0.97	0.995	0.17	0.88	88.7	
	99.84	0.37	0.980	0.09	0.74	97.2	
